# Retraction: Effect of harvest season on the nutritional value of bee pollen protein

**DOI:** 10.1371/journal.pone.0277688

**Published:** 2022-11-16

**Authors:** The PLOS ONE Editors

The *PLOS ONE* Editors retract this article [1] because it was identified as one of a series of submissions for which we have concerns about authorship, competing interests, and peer review. We regret that the issues were not addressed prior to the article’s publication.

SNAK did not agree with retraction. EKT, KAK, MJA, SAF, DMBS, and ESME either did not reply directly or could not be reached.
